# Sensory prediction on a whiskered robot: a tactile analogy to “optical flow”

**DOI:** 10.3389/fnbot.2012.00009

**Published:** 2012-10-22

**Authors:** Christopher L. Schroeder, Mitra J. Z. Hartmann

**Affiliations:** ^1^Department of Biomedical Engineering, Northwestern UniversityEvanston, IL, USA; ^2^Department of Mechanical Engineering, Northwestern UniversityEvanston, IL, USA

**Keywords:** rat, vibrissae, prediction, tactile flow

## Abstract

When an animal moves an array of sensors (e.g., the hand, the eye) through the environment, spatial and temporal gradients of sensory data are related by the velocity of the moving sensory array. In vision, the relationship between spatial and temporal brightness gradients is quantified in the “optical flow” equation. In the present work, we suggest an analog to optical flow for the rodent vibrissal (whisker) array, in which the perceptual intensity that “flows” over the array is bending moment. Changes in bending moment are directly related to radial object distance, defined as the distance between the base of a whisker and the point of contact with the object. Using both simulations and a 1×5 array (row) of artificial whiskers, we demonstrate that local object curvature can be estimated based on differences in radial distance across the array. We then develop two algorithms, both based on tactile flow, to predict the future contact points that will be obtained as the whisker array translates along the object. The translation of the robotic whisker array represents the rat's head velocity. The first algorithm uses a calculation of the local object slope, while the second uses a calculation of the local object curvature. Both algorithms successfully predict future contact points for simple surfaces. The algorithm based on curvature was found to more accurately predict future contact points as surfaces became more irregular. We quantify the inter-related effects of whisker spacing and the object's spatial frequencies, and examine the issues that arise in the presence of real-world noise, friction, and slip.

## Introduction

As an animal moves through the environment, the spatial and temporal gradients of sensory data it acquires are related through the velocity of its moving sensory surfaces. This relationship is represented by the “complete derivative” (Munson et al., 2009), and represents a mathematically inviolate description of information flow over moving sensory surfaces.

In the field of visual neuroscience, the complete derivative has been termed the “optical flow” equation. The optical flow equation relates spatial and temporal intensity (brightness) gradients to the velocity of the animal (Barron et al., 1994; Beauchemin and Barron, 1995; Horn and Schunck, 2003). Recent papers have formalized the idea of “tactile flow” to describe the flow of strain energy density across the hand (Bicchi et al., 2008; Scilingo et al., 2008), and bending moment (torque) at the whisker base across the rodent vibrissal (whisker) array (Gopal and Hartmann, 2007; Hartmann, 2009).

Notably, the optic and tactile flow equations are typically used by assuming that the animal makes use of spatial and temporal gradients of brightness (vision) or strain energy (tactile) to compute the velocity of its sensor array.

We recently proposed a complementary scheme: namely, if the animal already knows its own velocity, then it can use the complete derivative to predict future sensory data (Gopal and Hartmann, 2007; Hartmann, 2009). Computing the complete derivative at multiple spatial scales would allow the animal to predict the stimulus that it will measure in the next sensory instant and provide a mechanism to distinguish between externally-generated and self-generated motion.

In the present work, we used the rat vibrissal system as a model to examine the plausibility of using the complete derivative to predict upcoming sensory data. Rats actively brush and tap their whiskers (vibrissae) against objects to tactually explore the environment. During detailed exploration of objects, rats move their vibrissae rhythmically, between 5 and 25 Hz (Welker, 1964; Berg and Kleinfeld, 2003). During navigation behaviors, however, rats often hold their whiskers out in a relatively static position as they follow along a wall or tunnel.

The present work was designed to investigate tactile flow across the vibrissal array in this type of translational navigation behavior. We used both simulations and a 1 by 5 array of artificial vibrissae (hardware) to investigate the plausibility of using the complete derivative to predict upcoming whisker-object contact points.

## Methods

Our initial investigations were performed in simulation, and we then investigated the real-world issues that arise with implementation on a 1 × 5 array of robotic whiskers.

### Algorithms for the determination of radial distance, slope, and curvature

#### Radial distance determination using translations instead of rotations

Radial object distance is defined as the Euclidean distance between the base of a vibrissa and the point at which it makes contact with an object. Previous work has shown that an object's contour can be extracted by continuous rotation of a vibrissa against an object (Kaneko et al., 1998; Solomon and Hartmann, 2010). As the vibrissa “sweeps” against the object through a rotation, measurement of the bending moment (torque) at the vibrissa base permits the radial object distance to be continuously computed, and the object contour thereby inferred. This technique was empirically validated with sweeps of a vibrissa past three differently shaped objects (Solomon and Hartmann, 2010). The rotational sweep models vibrissal motion during typical “whisking” behavior, in which the rat rotates its vibrissae at their base.

During wall-following behavior, however, rats do not always whisk, but sometimes keep their vibrissae protracted against the wall. This behavior is better modeled as a relative translation between vibrissa and object, rather than as a rotation. Although Solomon and Hartmann (2010) developed equations to describe the translational sweep of a vibrissa past an object, the equations were not validated in hardware.

In the present study, we experimentally validate the translation equations developed in Solomon and Hartmann (2010) and use them to extract object contours. Equations 1–3 below provide only a brief overview of the differences between radial distance extractions during rotation versus translation. A more complete description of radial distance extraction based on rotation is provided in Solomon and Hartmann (2010).

As shown in previous work (Kaneko et al., 1998; Solomon and Hartmann, 2006, 2008, 2010, 2011; Birdwell et al., 2007) the radial distance *r*_0_ to the initial contact point on an object can be calculated using

(1) $$r_{0}=k\frac{\phi_{0}}{M_{0}}$$

where *k* = 3*EI*, *E* is the elastic modulus of the vibrissa, *I* is the area moment of inertia, ϕ_0_ is a small pushing angle beyond initial contact (typically about 3°), and *M*_0_ is the bending moment at the vibrissa base.

Once the radial distance *r*_0_ to the initial contact point is calculated, the whisker undergoes either a small rotation (*d*ϕ) or translation (*dL*). Radial distance at the current time step (*r*_*i*_) can then be calculated based on estimates of radial distance at previous time steps.

The procedures for calculating *r*_*i*_ after a translation or rotation are quite similar, but they differ in the calculation of the magnitude of the vector ${\bar{\delta }}_{i-1}$, perpendicular to the longitudinal axis of the vibrissa at the contact point. With the x-axis defined to be parallel to the vibrissa at *t* = *t*_0_, Solomon and Hartmann (2010) define ${\bar{\delta }}_{i-1}$ for rotation as:

(2) $${\bar{\delta }}_{i-1}=-r_{i-1}\cdot d\phi \cdot \left[\begin{array}{c} \sin\phi_{i-1}\\ \cos\phi_{i-1} \end{array}\right].$$

where *r*_*i*−1_ is the radial distance at the previous time step (before rotation), *d*ϕ is the incremental rotation, and ϕ_*i*−1_ is the angle between the vibrissa base at time *t*_0_ and the base at time *t*_*i*−1_ (after rotation).

In contrast, ${\bar{\delta }}_{i-1}$ for translation is defined by:

(3) $${\bar{\delta }}_{i-1}=\left[\begin{array}{c} 0\\ dL \end{array}\right].$$

where *dL* is the incremental linear movement between time steps. It is important to note that the base of each vibrissa is fixed perpendicular to the direction of linear movement for this definition of ${\bar{\delta }}_{i-1}$.

The value of ${\bar{\delta }}_{i-1}$ is then used to calculate the next radial distance *r*_*i*_, regardless of whether the whisker was translated or rotated. For a detailed explanation of this calculation, please refer to Figure 4 of Solomon and Hartmann (2010) and Equations 6–11 also in Solomon and Hartmann (2010).

#### Computing object slope and curvature from radial distances

**Figure 1:**
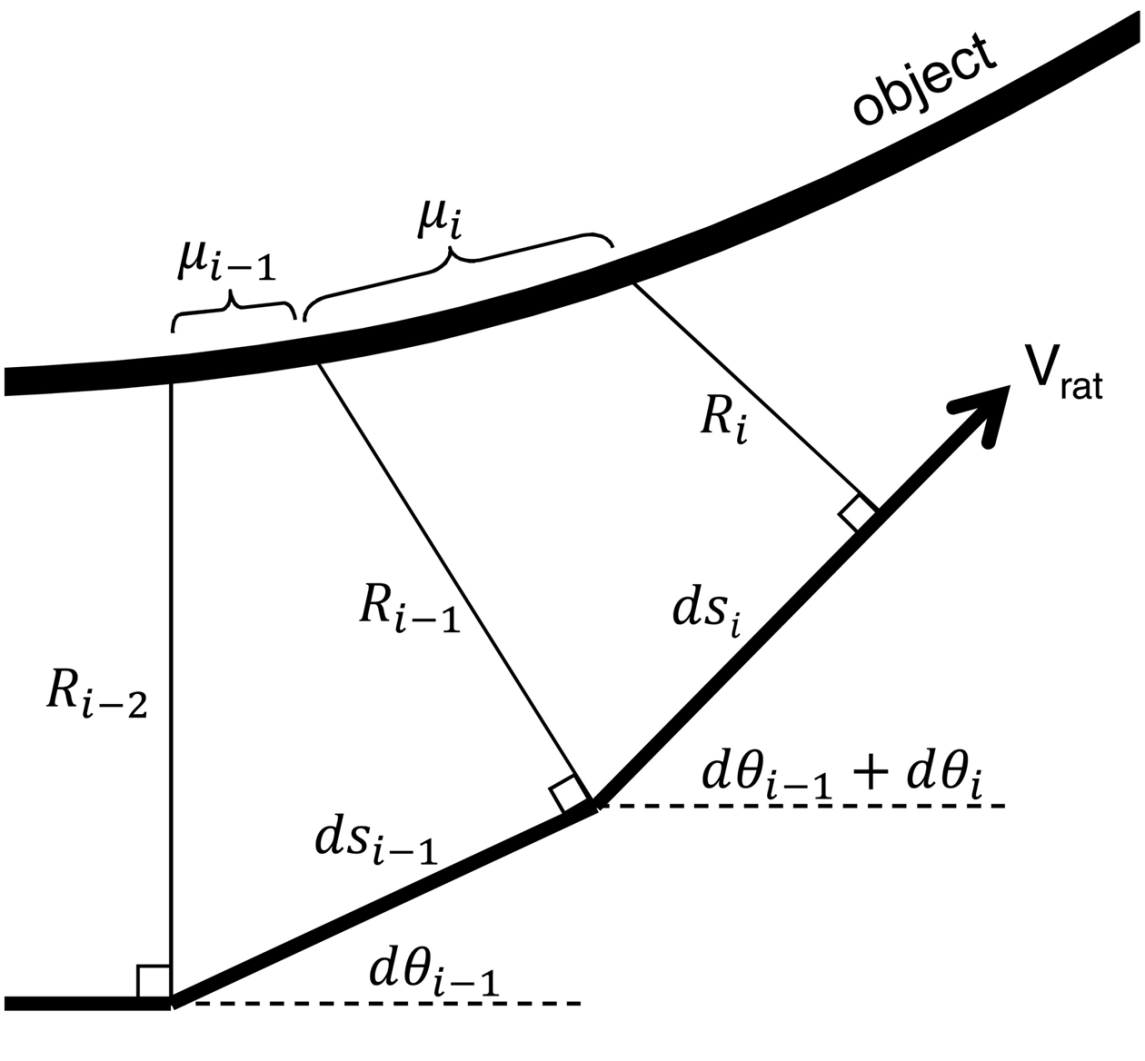
**Coordinate system for the determination of slope and curvature**. At each time step, radial distance is calculated relative to the object. The rat's velocity is indicated by *V*_rat_, and the arc *s* is always parallel to *V*_rat_. The distance *ds*_*i*_ is the distance in the direction of *s* between discrete time measurements *t*_*i*_ and *t*_*i*-1_. *ds*_*i*-1_ is the distance in the direction of *s* between discrete time measurements *t*_*i*-1_ and *t*_*i*-2_. *R*_*i*_ is the radial distance measure at the *i*^th^ time step, *d*θ_*i*_ is an angle measure of the change in direction of *V*_rat_, and μ_*i*_ represents the local slope of the object.

The local slope (μ) and the local curvature (κ) of an object can be calculated based on radial distance measurements. To calculate either μ or κ, we first define our coordinate system as shown in Figure 1. The distance *ds*_*i*_ is the distance the base-point of the whisker has translated in timestep *i*, and the direction of *ds* is always coincident with the rat's velocity. Radial distance estimates at multiple time points, the angle *d*θ, and the distance *ds* can then be used to find the slope μ_*i*_ at each time step:

(4) $$\mu_{i}=\frac{R_{i}-R_{i-1}+ds_{i}(d\theta_{i-1}+d\theta_{i})}{ds_{i}-R_{i}(d\theta_{i-1}+d\theta_{i})+R_{i-1}d\theta_{i-1}}$$

Slope μ_i_ is defined by the differences between radial distance measures (*R*_*i*_ and *R*_*i*−1_) divided by the distance traveled (*ds*_*i*_). Each *d*θ term accounts for changes in the rat velocity vector between radial distance measurements.

Calculation of curvature is similar, but requires us to first calculate the slope μ_*i*−1_ obtained in timestep *i*−1:

(5) $$\mu_{i-1}=\frac{R_{i-1}-R_{i-2}+ds_{i-1}d\theta_{i-1}}{ds_{i-1}-R_{i-1}d\theta_{i-1}}$$

Curvature is then defined using both μ_*i*_ and μ_*i*−1_:

(6) $$\kappa_{i}=\frac{\left|\frac{\mu_{i}-\mu_{i-1}}{ds_{i}}\right|}{{\left[1+{\left(\mu_{i}\right)}^{2}\right]}^{\frac{3}{2}}}$$

This equation is simply the discretized version of the definition of curvature measured in 2D Cartesian coordinates. Notably, the commonly-used simplification of Equation 6 that assumes μ_i_ is small compared to unity (and therefore reduces the equation to just the numerator) was empirically found to be inaccurate. The small slope assumption does not always hold in our calculations.

When the rat's heading does not change significantly between timesteps, *d*θ_*i*_ and *d*θ_*i*−1_ are both zero and Equation 4 can be simplified to:

(7) $$\mu_{i}=\frac{R_{i}-R_{i-1}}{ds_{i}}$$

where *R* and *s* still represent differences between radial distance measures (*R*_*i*_ and *R*_*i*−1_) over the distance traveled (*ds*_*i*_). Equation 5 can be similarly simplified and Equation 6 remains the same.

#### Calculating slope and curvature based on radial distances measured with multiple whiskers

**Figure 2:**
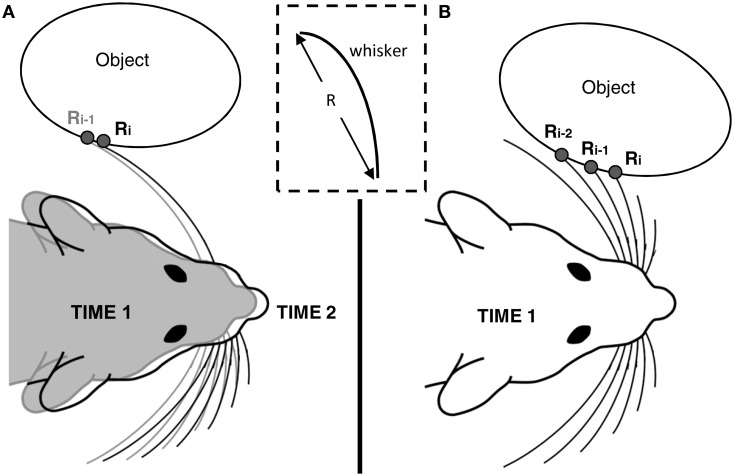
**Computing slope and curvature over multiple vibrissae**. The inset in the middle of the figure emphasizes that radial distance *R* is measured from the base of the whisker to the point at which the whisker contacts the object. **(A)** In the single whisker method, we imagine that the rat compares sensory data acquired from a whisker with the data it remembers having acquired on the previous timesteps. The rat shaded gray indicates the location of the rat at time 1, and the rat in black outline indicates the location of the rat at time 2, having translated slightly forward. **(B)** In the multiple whisker method, we imagine that the rat compares data across whiskers in the array at a single point in time. Both methods **(A)** and **(B)** can be used simultaneously to explore these different spatial scales in parallel.

So far, we have described the sensory data obtained by a single whisker over time. Equations 4–7 apply equally well, however, to multiple whiskers making simultaneous contact with an object, at different locations on the object. In this case, the subscript *i* in Equations 4–7 should be interpreted to mean the *i*^th^ whisker, instead of the *i*^th^ timestep. Figure 2 illustrates this idea. Figure 2A illustrates that calculation of slope and curvature with a single vibrissa requires memory of the data acquired on the previous timestep. In contrast, Figure 2B illustrates that calculation of slope and curvature with multiple vibrissa can be achieved within a single time step. This second method is equivalent to the rat integrating information across vibrissae within the array. In practice, calculation of slope and curvature can occur over both a single whisker (with memory) and multiple whiskers (at different spatial locations) simultaneously.

### Prediction of future points of contact and future curvature over two different spatial scales

Equations 1–7 define the calculations used to find radial distance, local object slope, and local object curvature. Using these calculated values, we now show that it is possible to make predictions about the sensory data the animal will receive.

#### “tactile flow” permits prediction of sensory data

Because animals control the movements of their limbs, they control the velocity with which sensory data flow over their sensory surfaces (Gopal and Hartmann, 2007; Bicchi et al., 2008; Nemenman et al., 2008; Scilingo et al., 2008; Hartmann, 2009). The time evolution of the flow field in space and time can be written via the complete derivative as shown in Equation 8.

(8) $$\frac{dA_{sen}}{dt}=\frac{\partial A_{env}}{\partial t}+V•\nabla A_{sen}$$

In this equation, *A* is any quantity that is being measured (intensity, temperature, etc.), and the vector *V* is the relative velocity between the sensory surface and the environment. The first term on the right represents intrinsic fluctuations in *A*, that is, changes in the environment. If environmental fluctuations are slow on the time scale of the animal's movements, that term becomes zero and the progression of gradients is almost completely deterministic. New information flows over the edges of the sensory surface, but thereafter, values may be computed by calculating the spatial gradients of *A* across the sensor. Equation 8 provides an inviolate mathematical description of information flow over moving sensory surfaces. The equation is not a model, it is necessarily true, in the same way that distance is equal to velocity integrated over time. Computing the terms of the complete derivative at multiple spatial and temporal scales would allow the animal to predict the stimulus that it will measure in the next sensory instant.

#### Interpreting “tactile flow” for an array of vibrissae

In this paper, we choose to represent “tactile flow” through radial distance of contact along the rat whisker because radial distance is directly related to changes in bending moment at the vibrissal base (Kaneko et al., 1998; Solomon and Hartmann, 2006). Prediction of future radial distances is possible by choosing radial distance (*R*) as the main parameter in the complete derivative shown in Equation 8. If the object being explored is static on the timescale of the animal's movements, then the first term on the right side of Equation 8 will be zero. In the context of the rat vibrissal system, we can therefore rewrite Equation 8 as follows:

(9) $$\frac{R_{i+1}-R_{i}}{\left(t_{i+1}-t_{i}\right)}=0+V\frac{R_{i}-R_{i-1}}{ds_{i}}\overset{\text{def}}{=}V\mu_{i}$$

where *R*_i+1_ is the radial distance of the future point of contact, *R*_*i*_ is the current radial distance, *t*_i+1_ and *t*_*i*_ are the times at which the radial distances are measured, *V* is the translational velocity of the rat, *ds*_*i*_ is the distance traveled in the last time step, and μ_*i*_ is the current slope. Rearranging the equation and substituting μ_*i*_ as in the last term above yields:

(10) $$R_{i+1}=R_{i}+V\mu_{i}(t_{i+1}-t_{i})$$

Thus it is clear that future radial distance can be estimated from the current slope.

If the slope of the object is changing, we expect the current curvature of the object to be able to give a better estimate of future radial distance. To use curvature, we first predict the future slope of the object, and then use that predicted slope to predict future radial distance. The future slope μ_*i*+1_ is found by choosing μ as the parameter in Equation 8, as shown in Equation 11:

(11) $$\frac{\mu_{i+1}-\mu_{i}}{\left(t_{i+1}-t_{i}\right)}=0+V\frac{\mu_{i}-\mu_{i-1}}{ds_{i}}$$

The simplification that $\kappa_{i}=\frac{\mu_{i}-\mu_{i-1}}{ds_{i}}$ was empirically found to generate significant error in our results. Therefore, in Equation 11 we replace $\frac{\mu_{i}-\mu_{i-1}}{ds_{i}}$ with the full equation for κ_*i*_ as shown in Equation 6, yielding:

(12) $$\mu_{i+1}=\mu_{i}+V\kappa_{i}\left(t_{i+1}-t_{i}\right)=\mu_{i}+V\frac{\left|\frac{\mu_{i}-\mu_{i-1}}{ds_{i}}\right|}{{\left[1+{\left(\mu_{i}\right)}^{2}\right]}^{\frac{3}{2}}}\left(t_{i+1}-t_{i}\right)$$

where μ_*i*+1_ is the local object slope at the future point of contact, μ_*i*_ is the current local object slope, *V* is the translational velocity of the rat, κ_*i*_ is the current local object curvature, and *t*_*i*+1_ and *t*_*i*_ are the times at which the radial distances used to calculate slope and curvature are measured. When Equation 12 is substituted into Equation 10 (with μ_i +1_ replacing μ_*i*_), we obtain an equation that estimates future radial distance based on both current slope and current curvature:

(13) $$R_{i+1}=R_{i}+V(\mu_{i}+V\kappa_{i}(t_{i+1}-t_{i}))(t_{i+1}-t_{i})$$

Finally, in the type of wall following behavior and environmental exploration in which prediction would be most useful to a rat, it is more likely that significant changes in calculated local object slope and local object curvature are due to measurement error than due to abrupt changes in the object. To decrease the sensitivity of predicted radial distance to measurement error, we average the local slope and curvature as follows:

(14) $$\mu_{\text{avg}}=\frac{1}{\left(N-1\right)}\sum_{i}^{N-1}\mu_{i}$$

(15) $$\kappa_{\text{avg}}=\frac{\text{1}}{\left(N-2\right)}\sum_{i}^{N-2}\kappa_{i}$$

where μ_avg_ is the average slope, κ_avg_ is the average curvature, *N* is either the number of past time steps (c.f., Figure 2A) or the number of vibrissae being used (Figure 2B), μ_*i*_ is the local object slope between each radial distance measurement, and κ_*i*_ is the local object curvature between each calculated slope value. Substituting Equations 14 and 15 into Equations 10 and 13 yields:

(16) $$R_{i+1}=R_{i}+V\mu_{\text{avg}}(t_{i+1}-t_{i})$$

and

(17) $$R_{i+1}=R_{i}+V(\mu_{i}+V\kappa_{\text{avg}}(t_{i+1}-t_{i}))(t_{i+1}-t_{i})$$

#### Simultaneous prediction with single and multiple vibrissae

As illustrated in Figure 2, local object slopes and curvatures can be computed either using a single whisker (with memory) or using multiple whiskers (distributed in space). For each of these two spatial scales, we can use either Equation 16 or Equation 17 to predict future contact points. We note that the use of multiple vibrissa permits the calculation of slope and curvature within a single timestep. In contrast, two or three timesteps are required to calculate slope and curvature using a single vibrissa. In addition, multiple vibrissae can be used to rapidly compute surface gradients on a larger spatial scale than would be possible with a single whisker. Using a single whisker would require memory of a duration equal to the inter-whisker-spacing divided by the velocity of the array, in order to obtain slope and curvature estimates at a spatial scale comparable to those obtained by multiple whiskers. Thus, there is a tradeoff between spatial scale and memory. Of course, computations can be done at both spatial scales (single and multiple whiskers) simultaneously.

### Hardware methods

Radial object distance is defined as the Euclidean distance between the base of a vibrissa and the point of object contact (Szwed et al., 2003; Birdwell et al., 2007). Previous work has shown that an object's contour can be extracted by continuous rotation of a vibrissa against an object (Solomon and Hartmann, 2010). As the vibrissa “sweeps” against the object through a rotation, measurement of the bending moment at the vibrissa base permits the radial object distance to be continuously computed, and the object contour thereby inferred. This technique was empirically validated with sweeps of a vibrissa past three differently shaped objects (Solomon and Hartmann, 2010). This same study demonstrated that a similar algorithm would work for translation of the vibrissa instead of rotation, but the translation technique was not empirically validated.

#### Vibrissa and vibrissa array design

**Figure 3:**
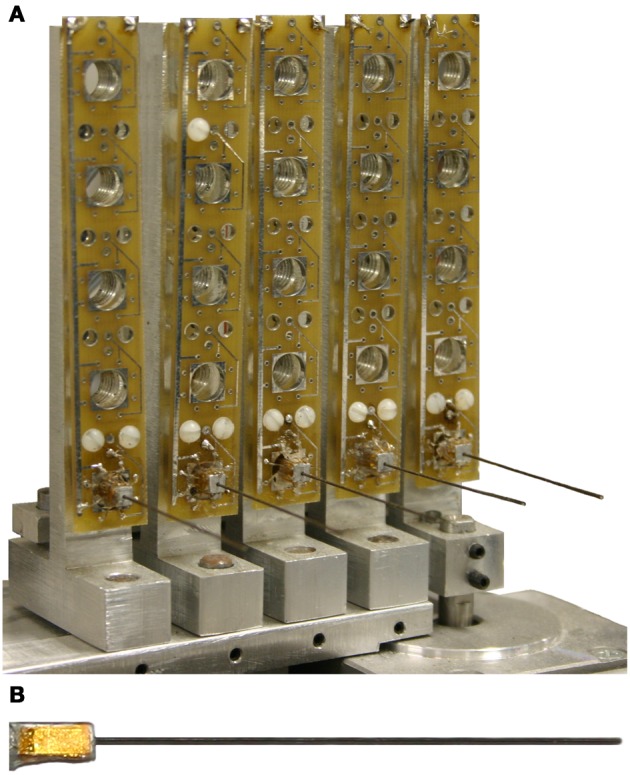
**(A)** 5 × 5 whisker array on individual rotating platforms and **(B)** individual whisker composed of 4 350 ohm strain gauges measuring the bending moment of a 0.020 diameter superelastic Nitinol wire.

The present work used a single horizontal row of a five by five array of vibrissae (Figure 3A). Vibrissae were constructed from Nitinol wire 4 cm in length and 500 μm in diameter. Nitinol wire was chosen because it is highly elastic and tends not to kink. Each wire was mounted in a 4 mm x 4 mm rectangular aluminum block with a strain gauge (Omega Engineering) attached to each block face (Figure 3B). This vibrissa design allows for a 2-D measure of strain, but in the present study the measured strain in the vertical plane was negligible compared to the strain in the plane of object translation (the horizontal plane).

#### Linear actuation

A linear actuation system was built to model the forward (translational) movement of a rat as it explores its environment. Because the vibrissa array was tethered with power and signal cables, it was easier to translate an object past the stationary vibrissa array than it was to translate the array past the object. In the present work, these two paradigms are equivalent because we are concerned only with relative velocity between the array and object. This study did not explore methods to distinguish between self-generated versus external movements.

**Figure 4:**
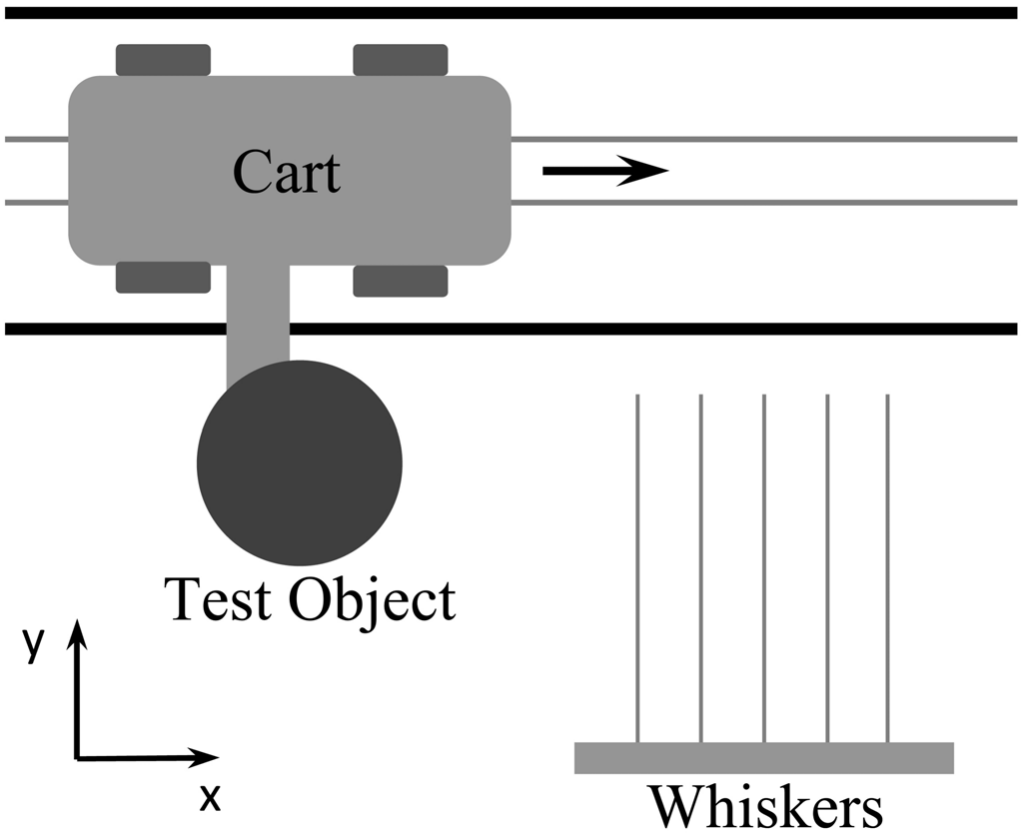
**Linear actuation system with test objects and artificial whiskers**.

As shown in Figure 4, a wheeled cart carried a test object along a track a fixed distance from the vibrissa array. The motion of the cart was controlled by an Animatics SmartMotor. The programmable motor controller and associated 4000 count/rev encoder allowed accurate position measurements within 10 μm. In practice, however, we only found it necessary to calculate position to within 0.1 mm. Similarly, velocity was calculated to within 0.1 mm/sec. These levels of accuracy were chosen because noise dominated the measurement error below these thresholds, causing there to be no appreciable difference in position measurement with increasing accuracy.

#### Device calibration

The two strain gauges for each sensing dimension on each vibrissa were arranged in a half Wheatstone bridge so the bending of the vibrissa created a change in voltage at the output of the circuit. The resting output was zeroed with a potentiometer. The actual value of the voltage output depended on circuit parameters such as gain, so it was necessary to calibrate the voltage output to the curvature at the base of the vibrissa. To calibrate the voltage to the curvature, the vibrissa was rotated against a peg placed at a known distance from the vibrissa base. For cylindrical homogenous vibrissae, curvature and moment differ only by a scaling factor. Given the known angular deflection of the cylindrical vibrissa and the distance between the base and peg, Solomon and Hartmann have shown that the moment can be calculated based on Euler-Bernoulli beam theory (Solomon and Hartmann, 2010).

Once a vibrissa has been calibrated, the voltage recorded from that vibrissa is converted to moment at the base of the vibrissa. The radial distance *r*_0_ to the initial contact point is calculated using Equation 1.

#### Error calculations in simulation and hardware

To quantify the accuracy of radial distance prediction using each method, prediction error was defined as:

(18) $$E_{pred}=\frac{1}{N}\sum_{i=1}^{N}\left|\frac{PRD_{i}-MRD_{i}}{MRD_{i}}\right|$$

where *E*_*pred*_ is the prediction error, *N* is the number of samples in a trial, *PRD*_*i*_ is the predicted radial distance for each sample, and *MRD*_*i*_ is the measured radial distance for each sample. *E*_*pred*_, which we define as prediction error, is the mean absolute error of the prediction. It is mean absolute percent error (MAPE) when multiplied by 100. For simulated results, *MRD*_*i*_ is chosen to be accurate to machine precision. This choice results in a prediction error that solely measures the accuracy of the prediction algorithm for a given set of parameters. In hardware, *MRD*_*i*_ is calculated using the measured bending moment *M* as shown in Equation 1. In this case, prediction error *E*_*pred*_ is affected both by measurement error as well as the accuracy of the prediction algorithm. In practice, however, errors in radial distance extraction (measurement errors) were small compared to errors in prediction.

## Results

As described in *Methods*, we use both Equation 16 (prediction using slope) and Equation 17 (prediction using curvature) to predict upcoming radial distance, and we use the two equations at different spatial scales.

### Prediction: simulation results

In all simulations in this section, we assume “perfect” radial distance extraction at time 1, and then calculate slope and curvature to predict radial distance at time 2. In other words, we do not simulate whisker deflection. The goal of this section is to verify that the use of either slope or curvature is sufficient for the prediction of future radial distance. Simulation will also show whether the increased mathematical complexity required by the use of local curvature leads to significantly increased predictive accuracy over the simpler equation for local slope.

In these simulations, the data collection method (i.e., single vibrissa or multiple vibrissa) is irrelevant, as the main difference between the two methods is spatiotemporal scale—the simulated objects can be made to arbitrary size and the sampling rate can be increased or decreased arbitrarily.

**Figure 5:**
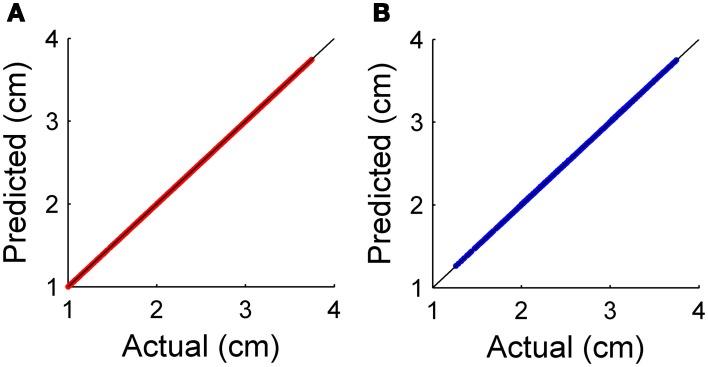
**(A)** Relationship between predicted and actual radial distance for prediction using local object slope **(B)** Relationship between predicted and actual radial distance for prediction using local object curvature.

To quantify prediction error from the use of Equations 16 and 17, we simulated translation of the whiskers past several differently sized cylinders. We then quantified how well the predicted values matched actual values. The results of this simulation are shown in Figure 5. As expected, both local object curvature and local object slope were good predictors of future radial distance, with error less than 0.1 ± 0.07% for each method. In these simulations, the error results only from discretization of slope and curvature.

**Figure 6:**
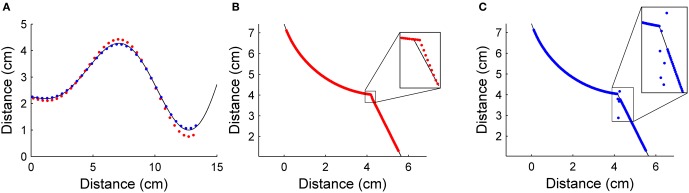
**(A) Radial distance predictions using both local object slope (red dots) and curvature (blue dots) for a shape with gradually changing curvature**. Prediction error is 6.37 ± 6.81% for local slope prediction and 3.02 ± 1.52% for local curvature prediction. It can be seen that local slope prediction leads to a slight overshoot when object curvature increases. **(B)** Equation 16 (prediction using slope) is used to predict radial distance for an object with an abrupt change in curvature (i.e., an edge). Average prediction error is 0.44 ± 0.12%. **(C)** Equation 17 (prediction using curvature) is used to predict radial distance for the same object as in **(B)**. Average prediction error is 1.05 ± 5.12%. In both **(B)** and **(C)**, the inset enlarges the area around the edge.

Figure 6 shows the results of simulating vibrissa contact with two different objects. Predictions about the future shape of the object were made using both Equations 16 and 17. The accuracy of these predictions for these simulated objects gives insight into the accuracy of each prediction method and shows the advantages and disadvantages of prediction using these two different equations.

In Figure 6A, local object slope and local object curvature were both used to make predictions about future contact points on an object that contained several gradual changes in curvature. Predictions using both slope and curvature were reasonably accurate over the object surface, with prediction errors of 6.37 ± 6.81% for prediction using local object slope and 3.02 ± 1.52% for prediction using local object curvature. In this first example, there is a clear advantage to choosing prediction using local object curvature. This simulation illustrates that prediction based on local object curvature is more accurate than prediction based on local object slope when curvature changes gradually.

The second test object (Figures 6B,C) had a location at which curvature abruptly changed—that is, an edge. Figure 6B shows that prediction using local object slope (Equation 16) acts similarly to a low pass filter with respect to change in curvature, resulting in a relatively accurate prediction. Figure 6C, in contrast, shows that prediction using local object curvature (Equation 17) was accurate until the abrupt change was reached, at which point there was a spike in the absolute percent error of the prediction. The prediction error of 0.44 ± 0.12% for prediction using local object slope was lower than the prediction error of 1.05 ± 5.12% for prediction using local object curvature.

In summary, these simulation results demonstrate that local object curvature more accurately predicts future contact points on regions of objects with no distinct edges, but that when objects have distinct edges, prediction using local object slope is more accurate. Of course, this in turn raises the question of what it means for an object to have a “distinct edge.” Mathematically, it must be an edge in the sense that there is a discontinuity in the curvature between measurements, which is clearly related to the spatial scale of the object relative to the spacing of the whiskers. We investigate this in the next section.

### Importance of whisker spacing

The spacing between the whiskers on the object surface places limits on the maximum curvature that can be sensed. Equation 19 defines κ_max_, the maximum discriminable curvature:

(19) $$\kappa_{\max}=\frac{1}{r}=\frac{1}{d+\Delta }$$

where *r* is the radius of the osculating circle defining κ_max_, *d* is the vibrissa spacing, and Δ is an arbitrarily small distance.

In the present work, we assumed that the spacing between the whiskers on the object surface was approximately equal to the spacing between them at their base. This is a reasonable approximation for the present work, in which the whiskers are parallel to each other, but is unlikely to be valid for the real rat.

**Figure 7:**
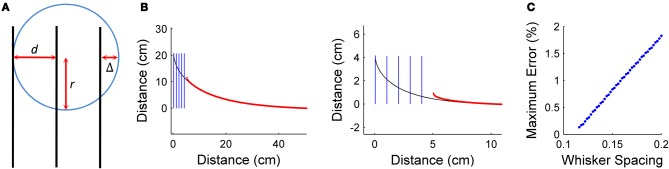
**Effect of vibrissal spacing**. **(A)** Maximum curvature discriminable by a given whisker spacing. **(B)** Prediction of radial distance on different curvature scales with 1 cm whisker spacing. The simulated object is shown in black, the starting positions of the vibrissae in blue, and the predicted contact points in red. The length of the spiral on the left is five times longer than the length of the spiral on the right. **(C)** Quantification of the increased prediction error as the scale of the object decreases relative to inter-vibrissal spacing. Whisker spacing is normalized by the length of the object. As relative vibrissal spacing increases, error increases linearly.

Figure 7A illustrates the relationship expressed in Equation 19. The distance Δ is added to the vibrissa spacing because a force must be applied to the vibrissae to find a contact point. The limiting case is finding the curvature of a cylinder with radius *r* and Δ = 0. With vibrissae spaced at *d* = *r*, the object could only apply a force to bend at most two of the vibrissae. In order to make a discrete curvature approximation, a minimum of three contact points are required to define curvature. Therefore, Δ must be strictly greater than zero if the measurement of curvature is desired.

To demonstrate the effect of relative vibrissa spacing on prediction error, prediction was simulated over hyperbolic spirals of various sizes. The hyperbolic spiral was chosen as the test object because it can be translated past the vibrissa array in such a way that curvature decreases approximately linearly. In these simulations, the multiple-vibrissa prediction method was used, with vibrissa spacing set to 1 cm. The size of the spiral test object was decreased for each case to illustrate the effect of relative vibrissa spacing.

Figure 7 shows the simulated test object on two different length scales, as vibrissa spacing stays constant. The left side of Figure 7B shows an object large on the scale of inter-vibrissa spacing, while the right side of Figure 7B shows an object small on the scale of inter-vibrissa spacing. Figure 7C illustrates that prediction error increases as the test object decreases in size. This increase in error is unsurprising, and occurs for two reasons. First, the relative change in distance between the most forward measurement and the predicted radial distance is more pronounced as the object decreases in size. More importantly, the spacing between vibrissae defines the resolution of the array, and it is decreasing relative to the change in curvature that is occurring. This simulation illustrates the importance of vibrissa spacing to prediction—though gradual curvature changes can be accurately predicted with good spatial resolution, prediction suffers when resolution decreases.

### Hardware results: validation of the translational sweep algorithm

**Figure 8:**
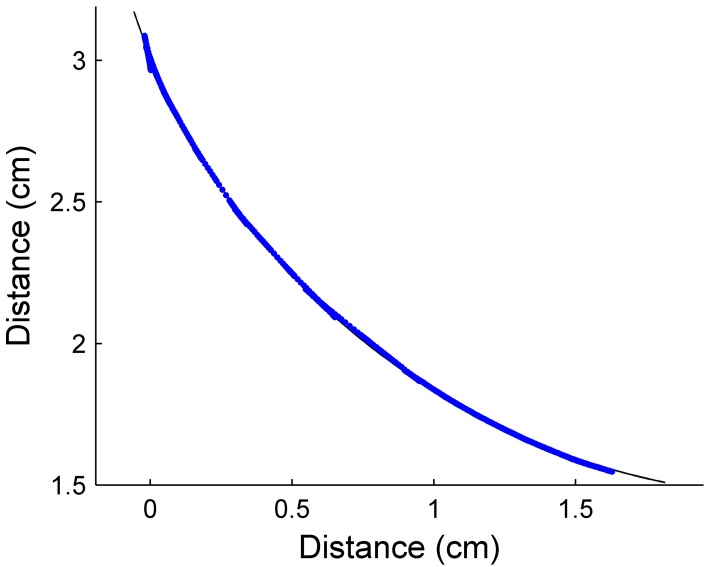
**Radial distance estimation, in hardware, using a translational sweeping algorithm to calculate radial distance**. Contour extraction was performed as a 2.5 cm diameter cylinder was translated past a single whisker. The estimated data points (blue) are plotted over a solid line (black) indicating the actual profile of the cylinder. The maximum difference between actual object position and the estimated contact point was 0.1 mm.

We next aimed to validate these simulation results in hardware. However, before we could do so, we needed to experimentally validate the translational sweep algorithm proposed by Solomon and Hartmann (2010). We implemented the translational sweep algorithm on our hardware array of whiskers (see *Methods*) to extract the contour of an object as it was translated past one or more vibrissae in the array. Figure 8 shows results from a trial in which a 2.5 cm diameter cylinder was translated past a single vibrissa at a velocity of 2 cm/s. The maximum difference between the actual object position and the estimated contact point at any given time was 0.1 mm, within the limits of measurement error.

### Hardware results: implementation of prediction with a single vibrissa: object of constant curvature

**Figure 9:**
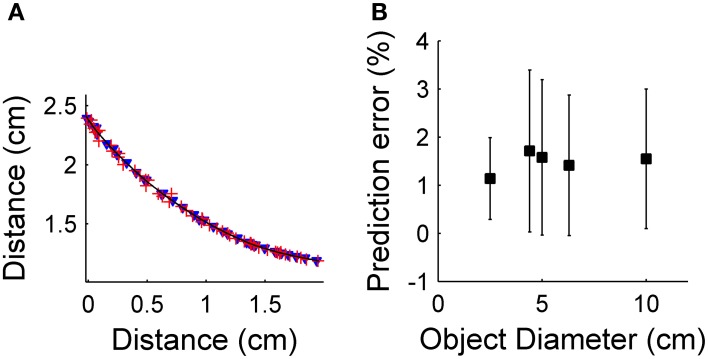
**(A)** Predicted radial distance plotted over a solid line indicating the actual profile of the cylinder. Predicted radial distances use local object curvature (blue) or local object slope (red). **(B)** Prediction error as the object diameter increases. There is no statistical difference between errors (*p*>0.05).

Having validated the translational sweep algorithm, we next aimed to test the two prediction methods in hardware. Figure 9A presents the same contour extraction data as in Figure 8, but here we also apply the prediction algorithms. Specifically, Figure 9A shows the contact points prediction using local object slope (Equation 16) and prediction using local object curvature (Equation 17). These predictions are plotted over a curve representing the actual surface of the cylinder. Using different numbers of past time steps did not significantly affect the accuracy of the prediction, which stayed fixed at an average level of 1.3 ± 1.25% for prediction using local object slope, and an average level of 1.1 ± 0.96% for prediction using local object curvature (Figure 9B). Prediction error was calculated according to Equation 18. Because there was no difference between numbers of past time steps used, five points were used for the rest of the single-vibrissa experiments presented in this paper. In the second part of this experiment, cylinders with diameters of 2.5 cm, 4.4 cm, 5.0 cm, 6.3 cm, and 10 cm were passed by the array at a velocity of 2 cm/s. As expected, there is no significant effect of object diameter on prediction error. The error across the trials for prediction using local object curvature was 1.40 ± 1.43%.

### Hardware implementation of prediction with multiple vibrissae: object of constant curvature

**Figure 10:**
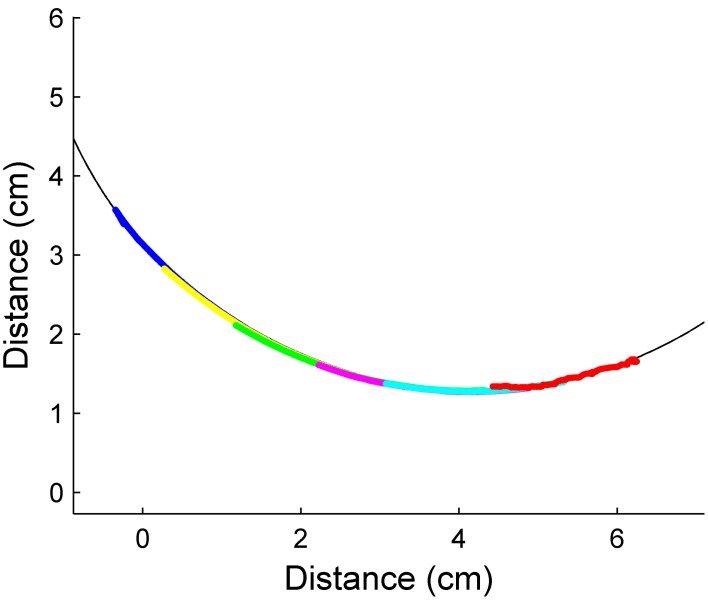
**Predicted radial distance using multiple whiskers**. The predicted future contact points (red) line up very well with the actual surface of the cylinder (black line). Prediction error is 1.13 ± 1.2%. Each different color corresponds to contact points from separate whiskers.

Neurons in the trigeminal nuclei have receptive fields that include multiple vibrissae, which may enable the rat to estimate the surface gradient at a single time point. For this technique to be viable, the chosen vibrissae must all be touching the object at once. The spacing between adjacent vibrissae in the final design of the array (Figure 3) is 1.5 cm. To ensure there were times when all five vibrissae touched the test object concurrently, only the 10 cm diameter cylinder was used for these constant curvature trials. Figure 10 shows the predicted future contact points for the vibrissa array as the array moves forward. Each predicted point is 1.5 cm further along the object than the lead vibrissa was at the time of the point's prediction. Since the five points of contact come from five different vibrissae, the predicted curvature gradient is implemented over a much larger spatial scale. Small errors in distance estimation result in larger deviations in the predicted future radial distance estimate.

### Hardware implementation of prediction with a single vibrissa: object with abrupt curvature change

The results of the previous two sections show that prediction using local object curvature can accurately predict future contact points for an object with constant curvature. In the world, however, such objects are rarely found. We did not implement prediction using the multiple vibrissa method on this object because the main difference between the spatial scales over which we were making abrupt changes was better represented by the single vibrissa method.

**Figure 11:**
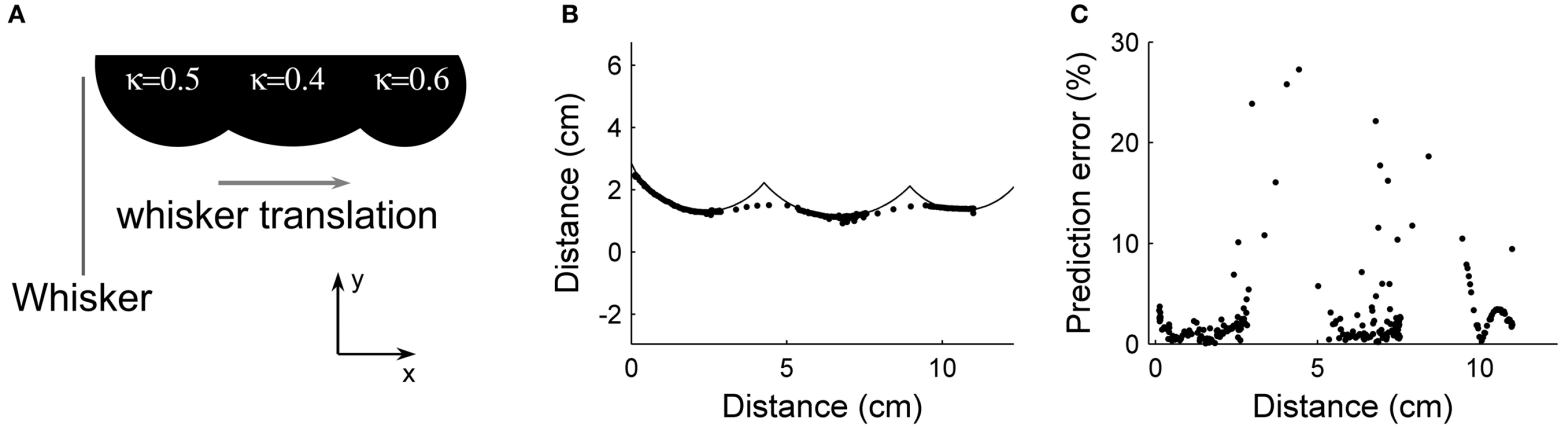
**Prediction of future contact points demonstrated in hardware using an object with abruptly changing curvatures**. **(A)** Test object, consisting of three circles placed in a row. The curvature of each circle is indicated; the curvatures at which the circles join is very high and was not quantified exactly. **(B)** Predicted contact points mapped onto the test object. At the transition between curvatures, the absolute percent error of the prediction increases substantially. **(C)** Absolute percent error of the prediction for each of the predicted data points.

To show the effect of abrupt changes in object curvature on prediction, a simple case was examined. Figure 11A shows the test object. The object has three distinct constant curvatures with no smoothing transition from one to the next. The radii of curvature for the sections were 2.5 cm, 3.2 cm, and 2.2 cm. The object was moved past the vibrissa array using linear actuation at a velocity of 2 cm/s. Prediction was performed using local object curvature for each vibrissa, with predicted contact points shown in Figure 11B.

It can be seen from Figure 11C that the absolute percent error of the prediction remains small during the first section of constant curvature. At the transition to the second curvature, the absolute percent error increases, with the maximum value more than quadrupling the prediction error during the first section. Once the vibrissa has been able to sense the second curvature for a short time, absolute percent error returns to baseline. The second transition is similar to the first. A closer examination of Figure 11B shows fewer predicted points during the times when the algorithm is least accurate. The reason for this is that the vibrissa slips quickly over the concavity in the object, covering the distance more quickly than during slip-free data collection. This longitudinal slip causes a significant increase in error in the estimation of distance.

### Hardware implementation of prediction with multiple vibrissae: object with gradual curvature change

We tested the hardware vibrissa array with a section of a hyperbolic spiral that had an approximately linear decrease in curvature as the object was translated past the whiskers.

**Figure 12:**
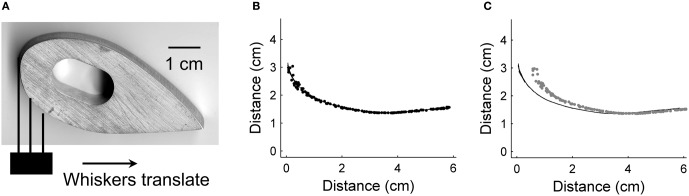
**Hardware prediction on an object with gradually changing curvature**. **(A)** Picture of the test object, designed to be a close approximation to a hyperbolic spiral. The whiskers translated along the bottom face of the object from left to right as shown by the arrow. **(B)** Spacing of contact points is small relative to object size, resulting in a prediction error of 0.97 ± 1.4%. **(C)** Spacing of contact points is large relative to object size, yielding a prediction error of 12.7 ± 7.11%.

Figure 12 shows the estimate of predicted future radial distance when the vibrissa array was passed by an object with gradually changing curvature. Figure 12A shows the actual object. Figure 12B shows a trial using the single vibrissa method, which corresponds to the situation where spacing between the data points is small relative to the change in curvature. The prediction error of 0.97 ± 1.4% is very similar to the error for an object with constant curvature (1.1 ± 0.96%). In Figure 12C, the vibrissa base points were evenly spaced at 1.5 cm intervals, and the multiple vibrissa method was used. This increase in spacing mirrored the decrease in size of the object in the simulations, and resulted in an increase in prediction error. Since the gradual change in curvature is from high curvature to lower curvature, the predicted future contact points lie mostly inside the actual curve. Prediction error for this trial was 12.7 ± 7.11%, though the error decreases to 11.9 ± 6.97% when the largest outlier is removed.

## Discussion

This paper has demonstrated that simple algorithms can be used to predict future contact points on an object. Prediction is accurate for objects that have constant and/or gradually changing curvature as long as the distance between vibrissae is small relative to the change in curvature. Abrupt changes in object curvature result in jumps in absolute percent error of the prediction.

### Prediction algorithm performance

Two different algorithms for prediction were described in the results section. Prediction using local object slope is only accurate when the curvature being measured does not change much between estimated contact points, but it can accommodate for abrupt changes in curvature (e.g., an edge). Prediction using local object curvature was shown to be more accurate for both objects with constant curvature and objects with gradual changes in curvature.

In all cases, vibrissa spacing makes a difference in accuracy of prediction. This result is hardly surprising, since the limiting factor is essentially sensor resolution. The main advantage of widely spaced vibrissae is the ability to predict further ahead in space. Since information about the sensed object is spread over a wider distance, it is more likely that the estimated curvature will be a reflection of the overall curvature of the object rather than a measurement of a local deviation from the actual object curvature.

These observations lead to the hypothesis that vibrissa spacing represents a trade-off between accuracy and predictive utility. For wall following, we anticipate that the rat will protract its vibrissae far forward and maximize spacing between the tips, because predictive sensing over a large spatial scale is important. For edge detection tasks, accuracy becomes more important so we anticipate that the vibrissal tips will be spaced more closely together.

### A computational mechanism for the instant detection of motion

Motion detection, as well as the ability to distinguish self-movement from environmental movement, is critical to animal survival. Behaviors such as escape or predation must link motion detection to immediate motor action. For these behaviors, the quality of the sensory data obtained is largely irrelevant, as long as it is sufficient to trigger the appropriate motor action. We suggest that a mismatch between predicted and actual sensory input may serve to direct attention.

In this work, we have presented specific examples of how calculating terms of the total derivative might be used by a moving rat to track an object within its vibrissal sensory array. If an object is moving in the vibrissal field, there will be a mismatch between actual and predicted input that is exactly equal to how the world is changing in time. In the next time step, the animal can use this mismatch to compute the relative velocity between its own movements and movements in the world. In other words, the animal can compare predicted and actual sensory data obtained to estimate the quantity $\frac{\delta A_{env}}{\delta t}$. Of course, if the fluctuations in the environment (i.e., movements of objects in the world) are unpredictable, then the animal will never succeed in finding an accurate estimate. Because objects on the scale of the rat are strongly dominated by inertial forces, however, many changes in the world will have predictable temporal trajectories (e.g., a rolling tin can).

### Advantages of using prediction during wall following behavior

During wall-following behavior, a rat maintains a small separation between itself and the wall while traveling at a relatively high velocity. The rat can maintain this separation even when walls curve, however, since the rat has mass, and it moves at a high velocity, changing direction takes time and energy. Following a curving wall would be easier if the rat could predict the future wall profile using its vibrissae. More specifically, if a rat could use just radial distance measures to predict the upcoming wall contour, it could start to change direction sooner, reducing inertial delays and saving energy. Encoding by its vibrissa array to determine where the wall was likely to be in the future, it would save energy and allow the rat to travel along the wall at a higher velocity. With these prediction algorithms that prediction could be accomplished at a very low level of processing. The algorithms presented here suggest that this type of prediction could be achieved with a very low level of computation.

### Possible improvements in vibrissa sensing/prediction

An examination of the results presented in Figure 11 shows predicted data points that are spaced much less densely in the concave regions of the object. Data are less dense in these sections because the vibrissa slips, and because only the tip of the vibrissa contacts during part of the movement. When the vibrissa tip is the only part of the vibrissa that contacts the object, radial distance cannot be accurately calculated with our algorithm. For artificial whiskers like the ones presented in this work, these sorts of tip contacts can occur at several radial distances near the full whisker length. In order to calculate radial distance for tip contacts, we need a measure of the axial force being applied to the vibrissa tip, where axial force is defined along the axis of the vibrissa. Future versions of the vibrissa array presented in this paper will be able to sense axial force along with bending moments at the vibrissa base.

The artificial whiskers used in this work were cylindrical, but real rat whiskers taper linearly. Tapering the whisker confers at least two advantages. First, whisker taper increases sensitivity to small contact forces (Williams and Kramer, 2010; Solomon and Hartmann, 2011). Second, when used in conjunction with an axial force sensor (not used in the present work), a tapered whisker ensures that the mappings between bending moment and axial force are one-to-one with radial distance and θ_*push*_, the angle through which the whisker has rotated against the object (Solomon and Hartmann, 2011).

### Conflict of interest statement

The authors declare that the research was conducted in the absence of any commercial or financial relationships that could be construed as a potential conflict of interest.

## References

[B1] Barron J. L. Fleet D. J. Beauchemin S. S. (1994). Performance of optical flow techniques. Int. J. Comput. Vis. 12, 43–77

[B2] Beauchemin S. S. Barron J. L. (1995). The computation of optical flow. ACM Comput. Surv. 27, 433–467

[B3] Berg R. W. Kleinfeld D. (2003). Rhythmic whisking by rat: retraction as well as protraction of the vibrissae is under active muscular control. J. Neurophysiol. 89, 104–117 10.1152/jn.00600.2002 12522163

[B4] Bicchi A. Scilingo E. P. Ricciardi E. Pietrini P. (2008). Tactile flow explains haptic counterparts of common visual illusions. Brain Res. Bull. 75, 737–741 10.1016/j.brainresbull.2008.01.011 18394519

[B5] Birdwell J. A. Solomon J. H. Thajchayapong M. Taylor M. A. Cheely M. Towal R. B. Conradt J. Hartmann M. J. Z. (2007). Biomechanical models for radial distance detection by rat vibrissae. J. Neurophsiol. 98, 2439–2455 10.1152/jn.00707.2006 17553946

[B6] Gopal V. Hartmann M. J. Z. (2007). Using hardware models to quantify sensory data acquisition across the rat vibrissal array. Bioinspir. Biomim. 2, S135–S145 10.1088/1748-3182/2/4/S03 18037723

[B7] Hartmann M. J. Z. (2009). Active touch, exploratory movements, and sensory prediction. Integr. Comp. Biol. 49, 681–690 10.1093/icb/icp107 21665850

[B9] Horn B. K. P. Schunck B. G. (2003). Determining optical flow. Artif. Intell. 17, 185–203

[B9a] Kaneko M. Kanayama N. Tsuji T. (1998). Active antenna for contact sensing. IEEE Trans. Rob. Autom. 14, 278–291

[B10] Munson B. R. Young D. F. Okiishi T. H. Huebsch W. W. (2009). Fundamentals of Fluid Mechanics. Hoboken, NJ: John Wiley and Sons, Inc

[B11] Nemenman I. Lewen G. D. Bialek W. Van Steveninck R. R. D. (2008). Neural coding of natural stimuli: information at sub-millisecond resolution. PLoS Comput. Biol. 4:e1000025 10.1371/journal.pcbi.1000025 18369423 PMC2265477

[B12] Scilingo E. P. Sgambelluri N. Bicchi A. (2008). The role of tactile flow in processing dynamic haptic stimuli in Sense of Touch and its Rendering: Progress in Haptics Research Book Series, eds Bicchi A. Buss M. Ernst M. O. Peer A. (Heidelberger, Berlin: Springer-Verlag Berlin), 39–60

[B13] Solomon J. H. Hartmann M. J. (2006). Robotic whiskers used to sense features. Nature 443, 525 10.1038/443525a 17024083

[B13a] Solomon J. H. Hartmann M. J. Z. (2008). Artificial whiskers suitable for array implementation: accounting for lateral slip and surface friction. IEEE Trans. Rob. 24, 1157–1167

[B14] Solomon J. H. Hartmann M. J. Z. (2010). Extracting object contours with the sweep of a robotic whisker using torque information.. Int. J. Rob. Res. 29, 1233–1245

[B15] Solomon J. H. Hartmann M. J. Z. (2011). Radial distance determination in the rat vibrissal system and the effects of Weber's law. Philos. Trans. R. Soc. Lond. B Biol. Sci. 366, 3049–3057 10.1098/rstb.2011.0166 21969686 PMC3172605

[B16] Szwed M. Bagdasarian K. Ahissar E. (2003). Encoding of vibrissal active touch. Neuron 40, 621–630 10.1016/S0896-6273(03)00671-8 14642284

[B18] Welker W. I. (1964). Analysis of sniffing of the albino rat. Behaviour 22, 223–244

[B19] Williams C. M. Kramer E. M. (2010). The advantages of a tapered whisker. PLoS ONE 5:e8806 10.1371/journal.pone.0008806 20098714 PMC2808387

